# Prognostic performance of age-adapted SOFA and qSOFA in septic children

**DOI:** 10.1186/s13054-019-2609-0

**Published:** 2019-10-29

**Authors:** Xuepeng Zhang, Xiying Gui, Kaiying Yang, Siyuan Chen, Yi Ji

**Affiliations:** 1 Department of Critical Care Medicine, West China Hospital of Sichuan University, Chengdu, 610041 China; 2 Department of Pediatric Surgery, West China Hospital of Sichuan University, #37 Guo-Xue-Xiang, Chengdu, 610041 Sichuan China

Dear editors:

The criteria used to define pediatric sepsis have not been updated for nearly 15 years since the establishment of the *2005 International Pediatric Sepsis Consensus*. Some investigators adapted the Sepsis 3.0 criteria to pediatric sepsis definition [1].

Between January 2018 and July 2019, we prospectively enrolled 342 children from PICU (Clinicaltrials.gov, NCT03598127) with sepsis on admission according to the *2005 Pediatric Sepsis Consensus*. Age-adapted SOFA and quick SOFA (qSOFA) were used as described in a previous study [1]. We assessed the performance of age-adapted SOFA and qSOFA, Pediatric Risk of Mortality (PRISM), and pediatric logistic organ dysfunction (PELOD)-2 scores on predicting mortality among septic children by using the area under the receiver operating characteristic curve (AUROC).

The median age was 9 months, and 192 children (56.14%) were boys (Table 1); 20 children died in hospital, with a mortality rate of almost 6%. Performances of the four tools on discriminating survival are showed in Fig. 1: the PELOD-2 scores and PRISM scores had good discrimination (0.871, 95%CI 0.831–0.905 and 0.868, 95%CI 0.828–0.902 respectively), better than age-adapted SOFA scores (0.790, 95% CI 0.743–0.832). Age-adapted qSOFA had the smallest AUROC (0.639, 95% CI 0.586–0.690).

**Table 1:** Baseline characteristics

Characteristics	Value
Age, months	9.00 (2.75, 42.0)
Male, *n* (%)	192 (56.14%)
Source of infection, *n* (%)	
Respiratory system	187 (54.68%)
Gastrointestinal system	81 (23.68%)
Central nervous system	13 (3.8%)
Bloodstream	33 (9.65%)
Other	28 (8.19%)
Length of ICU stay, days	7.90 (4.50, 13.60)
PRISM	8.0 (6.0, 13.0)
PELOD-2	4.0 (2.0, 6.0)
qSOFA	2.0 (1.0, 3.0)
SOFA	4.0 (3.0, 7.0)
Mortality, *n* (%)	20 (5.85%)

Data are presented as median (interquartile range) or number (percentage)

Abbreviations: *PICU* pediatric intensive care unit, *PRISM* Pediatric Risk of Mortality, *PELOD-2*, pediatric logistic organ dysfunction-2, *qSOFA* quick Sequential Organ Failure Assessment, *SOFA* Sequential Organ Failure Assessment

**Fig. 1:**
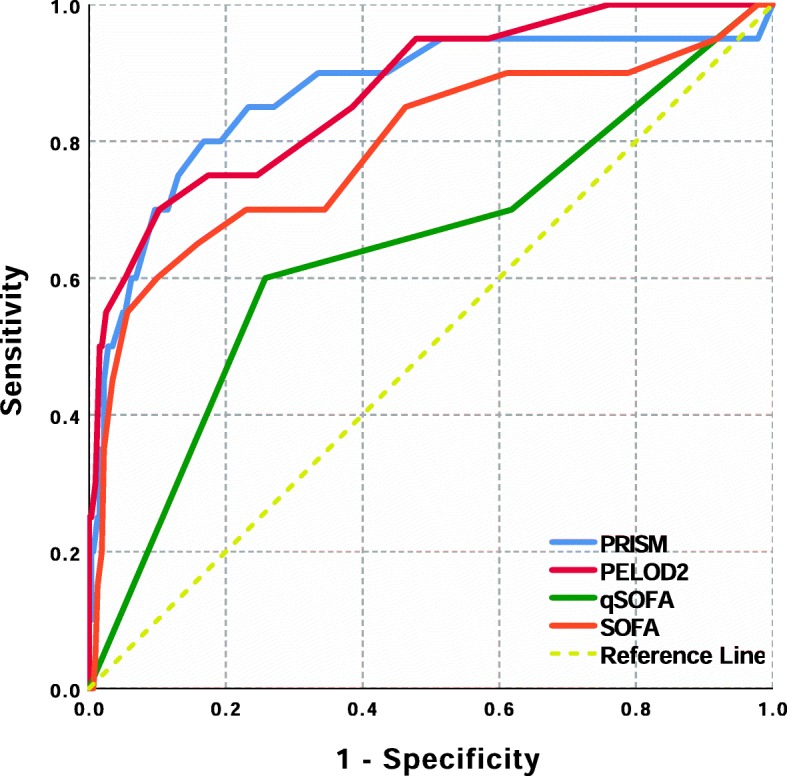
ROC for PRISM, PELOD-2, qSOFA, and SOFA scores

Our data revealed that age-adapted qSOFA may not be a good predictor of mortality for pediatric sepsis. Quick SOFA has a range from 0 to 3. It is possible that four variations may be insufficient to describe various severity of sepsis. In addition, scoring of Glasgow coma scale (GCS) score may be inaccurate in children, especially in young infants.

Age-adapted qSOFA may be sensitive in recognizing patients with sepsis and facilitates clinicians to quickly find out children who are at high risk for sepsis. However, when considering the limited specificity, promoting qSOFA in children with sepsis may have little benefit. It was reported that the mortality in pediatric sepsis was 3.5–4.4% [2, 3], which is similar to or even lower than the general mortality in PICU [4, 5] but much lower than the mortality in adult sepsis. Should we urge to adapt qSOFA to pediatric sepsis, in which the mortality is not higher than that in general ICU population? Maybe we could focus our attention on the patients with septic shock in whom the mortality is higher than 30%, as this may be more helpful to improve outcomes in children with sepsis.

## Data Availability

The datasets used for the analysis in the current study are available from the corresponding author on reasonable request.

## Abbreviations

PICU: Pediatric intensive care unit
PRISM: Pediatric Risk of Mortality
PELOD: Pediatric logistic organ dysfunction
SOFA: Sequential Organ Failure Assessment
qSOFA: Quick Sequential Organ Failure Assessment
CI: Confidence interval
AUROC: Area under the receiver operating characteristic curve
GCS: Glasgow coma scale
